# Dual Targeting of Cancer Cells with DARPin-Based Toxins for Overcoming Tumor Escape

**DOI:** 10.3390/cancers12103014

**Published:** 2020-10-16

**Authors:** Elena Shramova, Galina Proshkina, Victoria Shipunova, Anastasia Ryabova, Roman Kamyshinsky, Andrey Konevega, Aleksey Schulga, Elena Konovalova, Georgij Telegin, Sergey Deyev

**Affiliations:** 1 Shemyakin–Ovchinnikov Institute of Bioorganic Chemistry, Russian Academy of Sciences, Miklukho–Maklaya Street 16/10, 117997 Moscow, Russia; viktoriya.shipunova@phystech.edu (V.S.); schulga@gmail.com (A.S.); Elena.ko.mail@gmail.com (E.K.); telegin@bibch.ru (G.T.); deyev@ibch.ru (S.D.); 2 Prokhorov General Physics Institute, Russian Academy of Sciences, Vavilova Street 38, 119991 Moscow, Russia; nastya.ryabova@nsc.gpi.ru; 3 National Research Center “Kurchatov Institute”, Akademika Kurchatova pl. 1, 123182 Moscow, Russia; kamyshinskii@phystech.edu (R.K.); konevega_al@pnpi.nrcki.ru (A.K.); 4 Shubnikov Institute of Crystallography of Federal Scientific Research Centre ‘Crystallography and Photonics’ of Russian Academy of Sciences, Leninskiy Prospect, 59, 119333 Moscow, Russia; 5 Moscow Institute of Physics and Technology, Institutsky Lane 9, Dolgoprudny, 141701 Moscow, Russia; 6 Petersburg Nuclear Physics Institute Named by B.P. Konstantinov of National Research Centre “Kurchatov Institute”, Orlova Roscha 1, 188300 Gatchina, Russia; 7 Peter the Great St. Petersburg Polytechnic University, Politehnicheskaya 29, 195251 St. Petersburg, Russia; 8 The Institute of Molecular Medicine, I.M. Sechenov First Moscow State Medical University, 119991 Moscow, Russia; 9 Research Centrum for Oncotheranostics, Research School of Chemistry and Applied Biomedical Sciences, Tomsk Polytechnic University, 634050 Tomsk, Russia

**Keywords:** Barnase, liposomes, HER2, EpCAM, cancer therapy

## Abstract

**Simple Summary:**

Targeted therapy of solid tumors represents a great challenge because of heterogeneity of tumor-associated antigen expression. To overcome this obstacle we propose a dual targeting therapy based on protein preparations capable of recognizing different of tumor-associated antigens on a tumor cell producing a directed cytotoxic effect. The dual specific therapy of breast carcinoma-bearing mice using the designed preparations eliminates both the primary tumor and distant metastases. The mono-targeting therapy aimed at single tumor-associated antigen did not suppress metastases at all. The proposed approach can serve as a potential therapeutic strategy that surpasses mono-specific targeting strategies in the anti-cancer efficacy.

**Abstract:**

We report here a combined anti-cancer therapy directed toward HER2 and EpCAM, common tumor-associated antigens of breast cancer cells. The combined therapeutic effect is achieved owing to two highly toxic proteins—a low immunogenic variant of *Pseudomonas aeruginosa* exotoxin A and ribonuclease Barnase from *Bacillus amyloliquefaciens*. The delivery of toxins to cancer cells was carried out by targeting designed ankyrin repeat proteins (DARPins). We have shown that both target agents efficiently accumulate in the tumor. Simultaneous treatment of breast carcinoma-bearing mice with anti-EpCAM fusion toxin based on LoPE and HER2-specific liposomes loaded with Barnase leads to concurrent elimination of primary tumor and metastases. Monotherapy with anti-HER2- or anti-EpCAM-toxins did not produce a comparable effect on metastases. The proposed approach can be considered as a promising strategy for significant improvement of cancer therapy.

## 1. Introduction

Cancer is a second leading cause of death globally and one of the biggest challenges facing biomedical scientists. Despite the considerable progress made in targeted cancer treatment, the heterogeneity of solid tumor greatly limits precision oncology therapy. Dual targeting strategies applying targeting moieties recognized different receptors in tumor population can be a solution of this problem.

Tumor targeting with naked antibodies and antibody-drug conjugates has become an established strategy for cancer-related therapy in clinics, particularly if conventional therapies have failed [1,2]. However, antibodies have practical limitations due to their poor expression yield and aggregation tendency, at least for some constructs [3,4]. A solution might come from the use of alternative non-IgG binding scaffolds [5,6]. Due to their small size, high affinity to target and robust production scaffold proteins represent an attractive alternative to immunoglobulin proteins.

Previously designed ankyrin repeat proteins (DARPins), a novel class of non-IgG scaffolds based on naturally occurring ankyrin repeats [7] have been shown to bind to protein targets with specificity and affinity exceeding those of antibodies [7,8,9,10]. DARPins are small (13−20 kDa), highly soluble in water, very stable and lack cysteine residues. A number of DARPin molecules that specifically bind to different tumor-associated antigens, such as human epidermal growth factor receptor 2 (HER2) or the epithelial cell adhesion molecule (EpCAM) overexpressed in breast and ovarian cancer cells, have been developed [8,9,10,11,12]. 

The transmembrane protein HER2 is one of the most well-studied tumor markers; its overexpression represents a hallmark of many types of tumors associated with an increased risk of metastasis and resistance to chemotherapy [13]. The epithelial cell adhesion molecule (EpCAM) has also emerged as a promising structure for targeted therapy of solid tumors. EpCAM is expressed at low levels on basolateral cell surfaces of some normal epithelia [14]. In contrast, high levels of homogenously distributed EpCAM are detectable on cells of epithelial tumors [15,16], and its overexpression represents an independent prognostic marker for reduced survival in patients with breast and ovarian cancer [17,18]. 

As a tumor-targeting moiety for delivery of cytotoxic agents (e.g. toxic proteins), DARPins are widely used in modern research [19,20,21]. Toxins of bacterial origin are widely used as cytotoxic component in anticancer therapy [21,22,23,24,25]. In this work we use ribonuclease Barnase (Bn) from *Bacillus amyloliquefaciens* [26] and *Pseudomonas* exotoxin A secreted by gram-negative bacteria *Pseudomonas aeruginosa* with removed or inactivated human B-cell recognition sites (LoPE) [27]. The cytotoxic action of Bn is based on its high ribonuclease activity, LoPE irreversibly inhibits eukaryotic elongation factor eEF2 that leads to protein biosynthesis blocking in a cell.

Previously we reported a novel method for preparation of HER2-targeted liposomes (80–90 nm in diameter), each containing thousands of encapsulated protein molecules [28]. Here we use this approach for the preparation of liposomes loaded with RNAse Bn for anti-HER2 cancer therapy. We have shown that simultaneous treatment of animals with anti-EpCAM fusion toxin based on LoPE and HER2-specific liposomes loaded with Bn leads to concurrent elimination of primary tumor and metastasis.

## 2. Results

### 2.1. In Vitro Characteristics of Liposomes Loaded with Bn

Previously we have shown that liposomes functionalized with HER2-specific DARPin can be an effective vehicle for proteins delivery to cancer cells [28]. Here we used this method for the preparation of ligand-targeted liposomes comprising large quantities of encapsulated toxic protein for the treatment of HER2-positive cancer in vivo.

**Figure 1:**
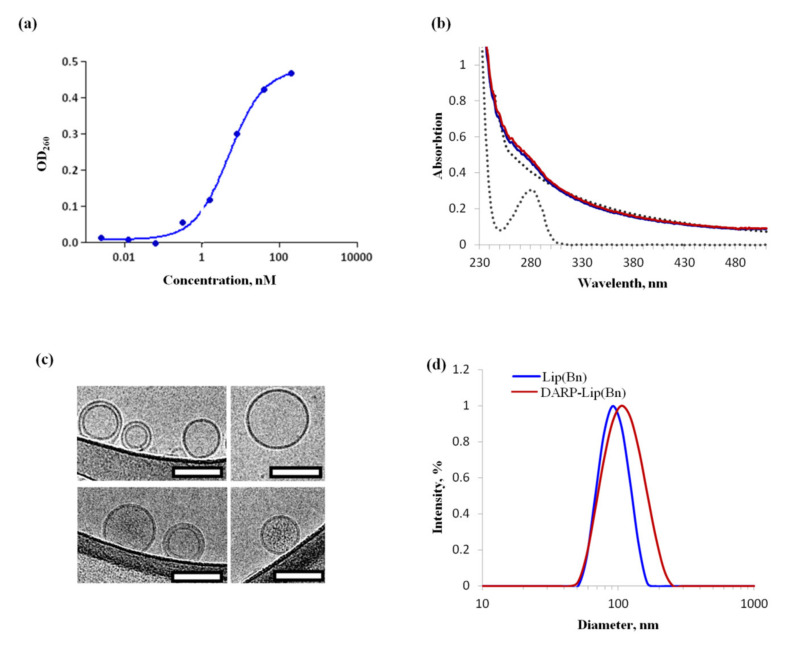
In vitro characteristics of liposomes loaded with Bn. (**a**) Ribonuclease activity of Bn. (**b**) Absorption spectroscopy of Bn-loaded liposomes (blue curve). Red curve is a sum of spectral curves corresponding to 6 µM Bn and 1 mg/mL liposomes (dotted grey curves). (**c**) Cryo-EM images of “empty” (upper panel) and Bn-containing liposomes (lower panel) at pH = 7.5. Scale bar = 100 nm. (**d**) Hydrodynamic size distribution by the intensity of DARP-Lip(Bn) and Lip(Bn) measured with the DLS technique.

The protein of non-immunoglobulin scaffold DARPin_9-29 recognizing HER2 with high affinity (K_D_ 3.8 nM) was used as a targeting module [11]. Ribonuclease Bn from *B. amyloliquefaciens* [29] was used as a cytotoxic moiety. *DARPin* and *Bn* genes were expressed in *Escherichia coli* BL21(DE3) strain as described by us previously [24,28]. RNAse activity of purified Bn was determined using the acid-insoluble RNA precipitate method [30]. The RNase activity of Bn was shown to be 68 ± 10% of that of the Bn standard (Figure 1a).

The encapsulation of Bn into liposomes is based on electrostatic interaction between proteins (positively charged at pH lower than pI) and a phospholipid membrane (charged negatively) at mild acidic pH. Extrusion of the proteolipid mixture through a 100 nm pore size membrane filter yielded unilamelar vesicles containing high quantities of proteins. The spectrum of Bn containing liposomes (Figure 1b, blue curve) can be fairly simulated (Figure 1b, red curve) by summing spectral curves corresponding to ~1.1 nM liposomes (concentration of the vesicles is 1 mg/ml suspension [28]) and 6 µM Bn (Figure 1b, dotted grey curves). The Bn to liposome molar ratio, which corresponds to the number of protein molecules in a liposome is thus equal to about 5454 (6000 nM/1.1 nM).

The morphology of the liposomes was assessed using cryo-EM. Cryo-EM images of both empty (Figure 1c, upper panel) and loaded with Bn (Figure 1c, lower panel) liposomes revealed unilamilar vesicles with round shape and distinctive lipid bilayer. As one can see from Figure 1c, the inside of the empty liposomes (the intra-liposome cavity) looks the same as the background, while some electron density is observed inside the Bn-containing vesicles. These observations are consistent with the very large number of Bn molecules that we succeeded to encapsulate per liposome.

Covalent coupling of DARPin_9-29 to the outer surface of the proteoliposome membrane included: modification of liposomes by 2-iminothiolane (Traut’s reagent), the reaction that introduces SH-groups to primary amine-containing phospholipids; modification of amino groups of the DARPin by sulfo-EMCS (N-ε-maleimidocaproyl-oxysulfosuccinimide ester), a hydrophilic amine- and sulfhydryl-crosslinker; coupling of the sulfo-EMCS-treated protein to the Traut’s reagent-treated liposomes.

Size and surface charge of DARPin-functionalized (DARP-Lip(Bn)) and non-functionalized (Lip(Bn)) proteoliposomes were measured by a dynamic light scattering (DLS) system (Zetasizer NanoZS, Malvern Panalytical, Malvern, UK). The diameters of Lip(Bn) and DARP-Lip(Bn) were equal to 90.91 ± 20.62 and 105.50 ± 15.96 nm, respectively (Figure 1d). ζ-potential of Lip(Bn) and DARP-Lip(Bn) were -10.7 ± 0.3 and -13.5 ± 1.5 mV respectively. A slight increase in size and decrease in ζ-potential prove the high efficiency of chemical conjugation of the target DARPin module with the liposome surface.

**Figure 2:**
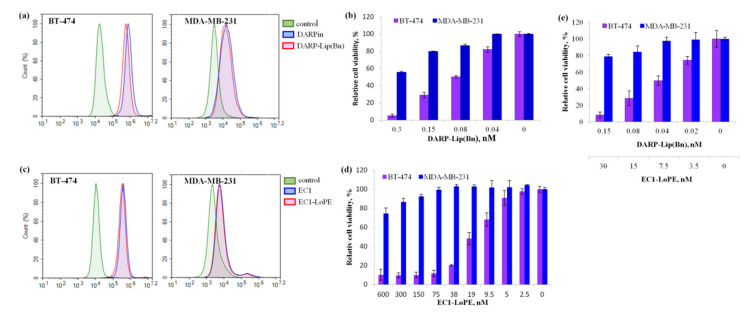
In vitro characteristics of DARP-Lip(Bn) and EC1-LoPE. (**a**,**c**) Normalized flow cytometry histograms showing specific interaction of DARP-Lip(Bn) and EC1-LoPE with HER2 and EpCAM receptors respectively. (**b**,**d**) In vitro cytotoxicity of DARP-Lip(Bn) and EC1-LoPE respectively. (**e**) Combined treatment of cells with DARP-Lip(Bn) and EC1-LoPE. Statistical analyses were performed using one-way analysis of variance (ANOVA). *p* < 0.01 versus control cells. Bars indicate SD for 3 independent experiments.

To prove that the proteoliposomes functionalized with DARPin_9-29 specifically interact with HER2 receptor on the cell surface, HER2-positive BT-474 cells stably expressing *NanoLuc luciferase* gene and MDA-MB-231 cells with a normal level of the HER2 expression were incubated with Cy3.5-labeled DARP-Lip(Bn) or DARPin as described in Materials and Methods. The results of the flow cytometry measurements indicate that the HER2-recognition property of DARPin in proteoliposomes complex is completely preserved: BT-474 cells treated with DARP-Lip(Bn) exhibit almost the same shift of the fluorescence intensity as the cells treated with DARPin as compared to untreated cells (~30- and ~40-fold respectively, Figure 2a). In MDA-MB-231 cells treated with dye-labeled conjugates in the same conditions, no significant fluorescence intensity shift in relative to the control was detected (Figure 2a).

The effect of DARP-Lip(Bn) on cell viability was determined by the cell cytotoxicity test (MTT). HER2-positive BT-474 cells as well as MDA-MB-231 cells with normal HER2 expression were incubated with nanomolar concentrations of DARP-Lip(Bn), for 72 h at 37 °C. As shown in Figure 2b the proteoliposomes strongly affected viability of HER2-positive cells. IC_50_ value estimated using nonlinear regression analysis was equal to 0.11 nM for BT-474 cells.

### 2.2. In Vitro Characteristics of EC1-LoPE

Another component used here for protein-based therapy is fusion protein based on EpCAM-specific DARPin (EC1) and domain I–truncated *Pseudomonas* exotoxin A with either removed or inactivated human B-cell recognition sites (LoPE). EpCAM, also known as CD326, is a 40 kDa type I membrane glycoprotein frequently expressed in human carcinomas, and involved in cell proliferation by linking to components of the Wnt signaling pathway and regulators of the cell cycle [16,31]. EpCAM attracts attention as a target for cancer-related immunotherapy due to its abundant expression in solid tumors, although its expression in normal epithelia is low [32,33].

Recombinant protein EC1-LoPE was purified as described in Materials and Methods and its functional activity was investigated in vitro. Flow cytometry has proved that EC1 preserved its EpCAM-recognizing ability within the fusion toxin: BT-474 cells treated with free EC1 exhibit almost the same shift of the fluorescence intensity as the cells treated with fusion toxin EC1-LoPE (Figure 2c). LoPE-mediated cytotoxicity in recombinant protein evaluated by MTT cytotoxicity test is also preserved and IC_50_ is equal to 15.3 nM (Figure 2d). 

To determine whether targeted liposomes DARP-Lip(Bn) and fusion toxin EC1-LoPE possess synergistic cytotoxic effect on HER2/EpCAM-positive cancer cells, we incubated BT-474 and MDA-MB-231 cells with different concentrations of DARP-Lip(Bn) and EC1-LoPE and assessed cytotoxicity after 72 h by MTT. As shown in Figure 2e, the viability of the HER2/EpCAM-overexpressing cell line treated with the combination of targeted cytotoxic agents was lower than with either single agent treatment. At the same time, the MDA-MB-231 cell line showed no decrease in cell viability after treatment with the combination of cytotoxic agents. Thus, combined treatment makes it possible to use lower concentrations of either of the toxic agents to reach the same level of cell death.

### 2.3. In Vivo Tumor Imaging, Tumor Distribution and Animal Treatment

To demonstrate the tumor-targeting capabilities of DARP-Lip(Bn) and EC1-LoPE in living animals, HER2/EpCAM–overexpressing tumor-bearing athymic BALB/c Nude mice were administered i.p. with proteoliposomes (10 nM) or fusion toxin (40 µg) conjugated with Cy5.5 as described in Materials and Methods, and then assessed by a living-animal imaging technique (using IVIS Spectrum CT, Perkin Elmer, Waltham, MA, USA) at different time points. 

**Figure 3:**
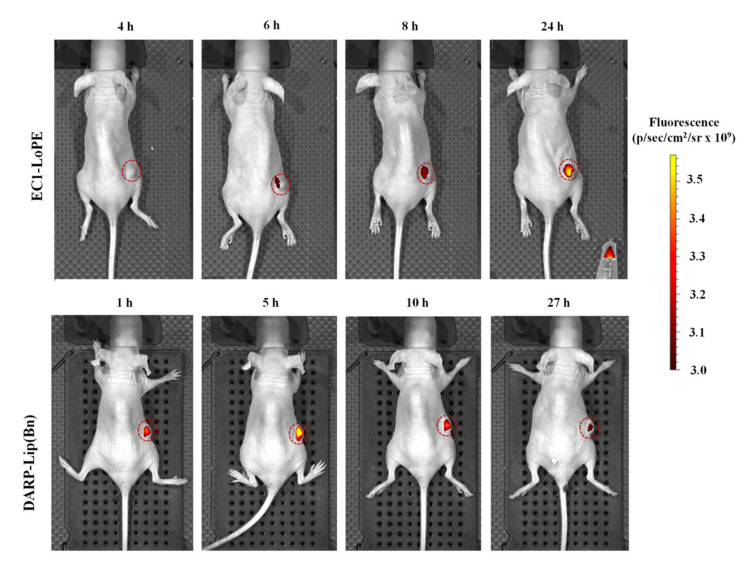
EpCAM/HER2-positive tumor imaging. Living animal photos confirming specific labeling of EpCAM (upper panel) or HER2 (lower panel)–overexpressing cells in vivo with EC1-LoPE or DARP-Lip(Bn).

As one can see from Figure 3, the dynamics of accumulation in the tumor is different for proteoliposomes and protein toxin. The highest tumor accumulation was observed at 24 and 5 h post-injection for EC1-LoPE and DARP-Lip(Bn) respectively (Figure 3). But in any case DARP-Lip(Bn) as well as EC1-LoPE are good markers for tumor imaging in vivo and displayed highly efficient tumor targeting.

**Figure 4:**
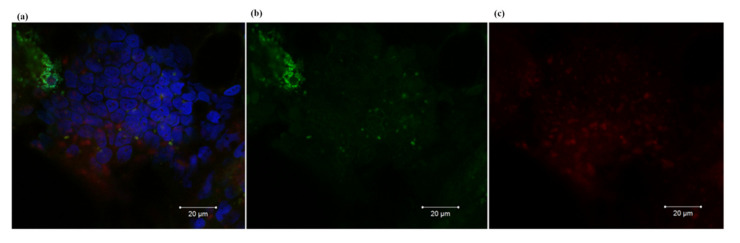
Microdistribution of DARP-Lip(Bn) and EC1-LoPE in the tumor. (**a**) Overlaid confocal images of tumor sections in blue (Hoechst 33342), green (Cy3) and red channel (Cy5.5) (Hoechst 33342: excitation femtosecond laser 740 nm, emission range 400–550 nm; Cy3: excitation laser 514 nm, emission range 550–630 nm; Cy5.5: excitation laser 561 nm, emission range 650–740 nm). (**b**,**c**) Confocal images of tumor sections in green and red channels respectively.

To investigate the distribution of DARP-Lip(Bn) and EC1-LoPE in tumor tissue in vivo, DARP-Lip(Bn) conjugated with Cy3 and EC1-LoPE conjugated with Cy5.5 were injected i.p. in HER2/EpCAM–overexpressing tumor-bearing athymic BALB/c Nude mice. Microscopic fluorescence images of the frozen sections of tissue samples were acquired 25 h after EC1-LoPE injection and 6 h after DARP-Lip(Bn) injection. DARP-Lip(Bn) (Figure 4b) and EC1-LoPE (Figure 4c) localized heterogeneously (according to chaotic blood vessel distribution) in the tumor. Intracellular localization of both target agents in the tumor with the distinct colocalization in the tumor area is observed (Figure 4a).

**Figure 5:**
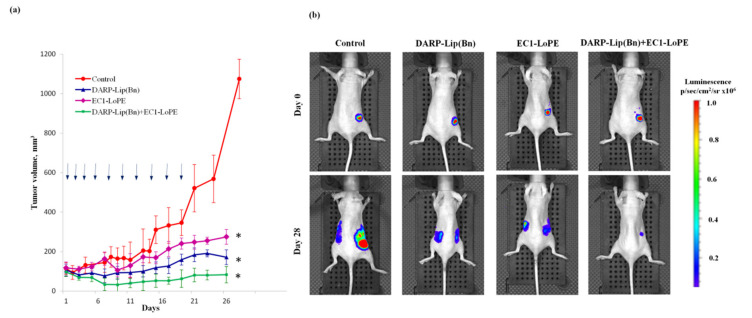
In vivo therapy and IVIS imaging: (**a**) Tumor growth dynamics upon the treatment with PBS, DARP-Lip(Bn), EC1-LoPE or DARP-Lip(Bn) plus EC1-LoPE. Arrows indicate the time of injection; bars indicate SD; * *p* < 0.05; (**b**) Imaging of BT474-NanoLuc tumor xenografts at the beginning (day 0) and at the end (day 28) of treatment. Mice were injected with 7 µg of furimazine and bioluminescence was recorded with IVIS Spectrum CT.

To investigate the anticancer potential of proteoliposomes for monotherapy, as well as for the combined therapy, BT-474/NanoLuc tumor-bearing mice were used. After tumor volume reached approximately 100 mm^3^, twenty mice were randomly divided into four groups and treated with DARP-Lip(Bn) only, DARP-Lip(Bn) plus EC1-LoPE, EC1-LoPE only, or PBS only. Tumor volumes and body weights were recorded every other day. No significant changes in body weight were registered during the treatment. As presented in Figure 5a, groups treated with DARP-Lip(Bn) or EC1-LoPE showed a significant inhibition of tumor-growth with TGI equal to 84% and 74.5%, respectively. The group of mice subjected to a combined treatment with DARP-Lip(Bn) and EC1-LoPE revealed the impressive result with TGI equal to 91.8%. Tumors in the control groups displayed a rapid growth and on day 28 after the treatment the tumor volumes in the control groups were approximately 9 times larger than the tumor volumes at the start of the experiment (Figure 5a).

The effectiveness of the treatment was also monitored using the IVIS Spectrum CT bioimaging system (Perkin Elmer, Waltham, MA, USA). Mice were injected i.p. with NanoLuc substrate furimazine and images were taken immediately. On day 28 of the treatment animals in the control group had large primary tumors as well as secondary tumor nodes growing in the area of the symmetric lymph node (Figure 5b). Although in animals treated with either DARP-Lip(Bn) or EC1-PE40 primary tumors were smaller than in the control group, luminescence imaging revealed distant metastasis in both groups. In contrast, animals subjected to combined therapy (DARP-Lip(Bn) plus EC1-LoPE) had only minor primary tumors and did not exhibit distant metastases (Figure 5b). 

## 3. Discussion

Targeting therapy of solid tumors represents a great challenge because of heterogeneity of tumor-associated antigen expression. Combination therapy, targeting two (or more) different receptors on a tumor cell has gained considerable attention in the field of oncology in recent years, with numerous studies demonstrating its significant advantage over monotherapies [34,35,36,37]. 

High levels of the HER2 expression observed in a variety of carcinomas and clinical success of the HER2-directed monoclonal antibody Herceptin make the HER2 gene product a promising therapeutic target [38]. EpCAM is also a promising therapeutic target, as numerous studies indicate its role as a cell surface marker for various types of carcinoma. A clinical study has shown that coexpression of both HER2 and EpCAM at high levels correlates with poor prognosis in breast cancer patients [39], suggesting that a drug targeting both these moieties would be highly beneficial to these high-risk patients.

In the present study we compared antigen-monospecific therapy with dual-specific therapy based on DARPin-fusion toxic complexes targeted two different tumor-associated antigens, HER2 and EpCAM. For this purposes we developed HER2-specific liposomes loaded with a large amount of Bn and created EpCAM-specific fusion protein based on DARPin and LoPE (Figure 1). 

The effectiveness of the developed targeted agents for solid tumors as well as metastasis treatment was evaluated using mouse model with xenograft tumors derived from BT-474 cell line. It was recently shown that ductal carcinoma cell line BT-474 has a great potential as cancer model with spontaneous metastasis [40,41,42,43]. It was shown BT-474-based tumors can form metastases in lymph nodes, lungs or spleen with various frequencies. Here we observed the formation of metastasis in inguinal lymph node after s.c. injection of BT-474 cells in all experimental animals that do not receive any specific treatment, which makes this tumor model a very convenient tool for tumor and metastasis grow dynamics study.

Antigen specificity and cell toxicity of these protein complexes were thoroughly proved in vitro (Figure 2). Using whole-body fluorescence imaging we demonstrated that DARP-Lip(Bn) and EC1-LoPE displayed highly efficient tumor targeting (Figure 3). Fluorescence histochemistry of the frozen sections of tissue samples revealed intracellular localization of both target agents in the tumor with the distinct colocalization in the tumor area (Figure 4). We have shown that monotherapy with EpCAM-specific LoPE or HER2-specific liposomes loaded with Bn significantly reduced primary tumors but did not affect distant metastases (Figure 5). On the other hand, dual-specific therapy led to elimination of both primary tumors and metastases.

Dual targeting strategy is widely used in contemporary oncology researches. It includes dual targeting of different epitopes on one receptor or ligand, dual targeting of a receptor and a ligand, dual retargeting of toxins (reviewed in [44,45,46]). Results from all these studies demonstrate that in general dual targeting strategies outperforms monotreatment. 

The results obtained in this work confirm the advantages of dual targeting therapy, and we can state that protein therapeutics for dual targeting suggests a new way in cancer treatment and will enter clinical study in the near future.

## 4. Materials and Methods 

Unless otherwise stated, reagents and chemicals were obtained from Sigma-Aldrich (St. Louis, MO, USA) and were used without further purification.

### 4.1. Proteins

The following proteins were used in experiments. DARPin_9-29 were produced in *E. coli* and purified as described in details in our previous work [28]. Wild-type Bn was extracted and purified from culture media following the method of Hartley [26] with slight modifications. 

Recombinant EC1-LoPE was produced in *Escherichia coli* BL21(DE3) strain cells transformed with the plasmids pET22-EC1-LoPE. The last plasmid was obtained by replacement of *Darpin_ 9-29* gene in the plasmid pDARP-LoPE [47] with the gene for *Darpin-EC1*. The Darpin-EC1 nucleotide sequence was deduced from its amino acid sequence published by Stefan et al. [12], taking into account the codon usage in highly expressed *E. coli* genes [48]. The *EC1* gene was assembled from chemically synthesized overlapped oligonucleotides of 50 bp length by PCR and placed into the plasmid pDARP-LoPE between restriction sites *Nde*I and *EcoR*I. Expression and purification procedures for EC1-LoPE were essentially the same as was described earlier for the protein DARPin-LoPE [47]. Briefly, *E. coli* was grown in autoinduction ZYM-5052 medium prepared according to Studier [49] containing 100 µg/ml kanamycin at 25 °C. The cells were harvested by centrifugation at 10,000 × g at 4 °C for 20 min, and resuspended in lysis buffer (200 mM Tris-HCl, 500 mM sucrose, 1 mM EDTA (pH 8.0), 1 mM PMSF and 60 µg/ml lysozyme). The suspension was diluted 2-fold with distilled water and incubated at room temperature for 30 min. Cells were broken on ice using a Vibra Cell ultrasonic liquid processor VCX130 (Sonics & Materials, Inc., Newtown, CT, USA). The cellular debris was pelleted at 70,000 × g at 4 °C for 30 min. After addition of imidazole (30 mM) and NaCl (500 mM), the supernatant was filtered through a 0.22 µm membrane and applied onto a HisTrap HP 1 ml column (GE Healthcare, Little Chalfont, UK) equilibrated with 20 mM NaPi (pH 7.5), 500 mM NaCl and 30 mM imidazole. The bound proteins were eluted with a linear 30–500 mM imidazole gradient. The fraction containing EC1-LoPE was combined, diluted 5-fold with 25 mM Tris-Cl (pH 8.0), and loaded onto a MonoQ 10/100 GL column (GE Healthcare, Little Chalfont, UK) equilibrated with the same buffer. The bound proteins were eluted with a linear 0–1 M NaCl gradient. The fractions were analyzed by 15% reducing SDS-PAGE. Protein concentration was determined by UV spectroscopy using ε_280_ = 48,220 M^−1^ cm^−1^. 

### 4.2. Preparation of Bn-Encapsulated Liposomes

The phospholipid suspension was prepared by swelling 0.2 g of L-α-phosphatidylcholine granules (Soy 40%, 341602, Avanti Polar Lipids, Alabaster, AL, USA) in 10 mL of distilled water for 16-20 h at ambient temperature. The suspension was further filtered through a 0.8 µm syringe filter and stored at 4 °C until used. 

An aliquot of 0.5 mL of the phospholipid suspension was mixed with 0.5 mL of Bn (4 mg/mL) in 20 mM K-Pi (pH 7.5). The protein-lipid suspension was frozen and thawed 5 times and subsequently extruded 19 times through a polycarbonate membrane with 100 nm pore size at ambient temperature using an Avanti Mini Extruder (Avanti Polar Lipids, Alabaster, AL, USA). The extrusion yields unilamelar liposomes (SUVs) with an average diameter of 80–90 nm. The liposomes were purified from the excess of Bn on a Sepharose CL-2B column (10 × 35 mm) equilibrated with 10 mM K-Pi (pH 7.5) containing 0.2 M NaCl. At high ionic strength the protein dissociates from the outer surface of the liposome membrane. Chromatography on a Sepharose CL-2B column enabled to completely separate the liposomes from the protein not associated with the vesicles. The liposome fraction, collected in the void volume, was stored at 4 °C until used. 

### 4.3. Covalent Coupling of DARPin_9-29 to Liposomes 

Covalent attachment of DARPin_9-29 to the outer surface of the liposome membrane was described in details in [28]. In short, liposomes containing Bn were prepared as described above and incubated with 6 mM 2-iminothiolane (Traut’s reagent) at ambient temperature for 1 h in 100 mM K-Pi (pH 8.0) containing 1 mM EDTA. Incubation with Traut’s reagent leads to the attachment of a residue terminated by SH-group to primary amino groups of the amine-containing phospholipids composing the liposome membrane. The phospholipid mixture used here (soy PC, 40%, from Avanti Polar Lipids, Alabaster, AL, USA) consisted of about 16% phosphatidylethanolamine, making it possible to introduce many SH-groups to the surface of a liposome. The incubation mixture was passed through a NAP-5 desalting column (GE Healthcare, Little Chalfont, UK) equilibrated in 100 mM K-Pi (pH 7.5). The void volume fraction (~0.5 mL) was collected and kept on ice.

DARPin_9-29 (2–4 mg/mL) was incubated with a 10-fold molar excess (1–2 mM) of sulfo-EMCS (N-ε-maleimidocaproyl-oxysulfosuccinimide ester) for 45 min under ambient conditions. This heterobifunctional amine-to-sulfhydryl linker, containing the succinimide (which specifically reacts with primary amino groups) and the maleimide (which reacts with thiols) moieties at opposite ends of the molecule, covalently binds to DARPin_9-29 through one of primary amino groups on the surface of the protein. The protein lacks SH-groups, thus leaving the maleimide group of the linker untouched and available for further reaction with the Traut’s reagent-treated liposomes. The DARPin modification should occur simultaneously with treatment of the liposomes with Traut’s reagent in order to avoid oxidation of SH-groups introduced to the bilayer. The incubation mixture was passed through a NAP-5 desalting column (GE Healthcare, Little Chalfont, UK) equilibrated in 100 mM K-Pi (pH 7.5). The sulfo-EMCS-treated DARPin was mixed with equal volume of the Traut’s reagent-treated liposomes and incubated for 2 h under ambient conditions. The non-bound DARPin was separated from the liposomes on a Sepharose CL-2B column (10 × 35 mm) equilibrated with 20 mM K-Pi (pH 7.5). The void volume fraction was collected and stored at 4 °C until used. 

### 4.4. Cryo-Electron Microscopy 

Cryo-electron microscopy (cryo-EM) was used for direct visualization of liposomes. Prior to cryo-EM study 3 μl of liposome suspension were applied to a glow discharged Lacey carbon EM grid, which was then blotted for 2.5 s at 4 °C and vitrified by a rapid plunging into liquid ethane pre-cooled with liquid nitrogen using Vitrobot Mark IV (ThermoFisher Scientific, Waltham, MA, USA). The obtained samples were studied using Titan Krios 60-300 TEM/STEM (ThermoFisher Scientific, Waltham, MA, USA) cryo-EM, equipped with XFEG electron source, TEM direct electron detector Falcon II (ThermoFisher Scientific, Waltham, MA, USA) and Cs image corrector (CEOS, Heidelberg, Germany) at accelerating voltage of 300 kV. To minimize radiation damage during image acquisition low-dose mode in EPU software (ThermoFisher Scientific, Waltham, MA, USA) was used.

### 4.5. Labeling with Fluorescent Dyes 

Fluorescent dyes were obtained from Lumiprobe (Lumiprobe, Hunt Valley, MD, USA). For the sake of visualization, DARPin, EC1-LoPE or proteoliposomes complexes (DARP-Lip(Bn)) lacking visible fluorescence were covalently labeled with Cyanine 3.5 (N-hydroxysuccinimide ester) dye. For the whole-body fluorescence image Bn or EC1-LoPE were conjugated with Cyanine 5.5. For the fluorescence histological analysis before incorporation into liposomes Bn was conjugated with Cy3 and EC1-LoPE—with Cy5.5.

For the labeling with cyanine dyes, a few grains of the dye were dissolved in water and the concentration of the solution was determined spectophotometrically. Eight-fold molar excess of the dye solution was used for conjugation with protein or proteoliposomes complexes. The reaction was carried out in 0.1 M phosphate buffer (pH 8.5) for 1 h at room temperature to get the dye-conjugate (protein or proteoliposome). The dye-labeled conjugate was separated from the unbound dye on NAP-5 desalting column (GE Healthcare, Little Chalfont, UK) equilibrated in 0.1 M phosphate buffer (pH 7.5). The void volume fraction was collected and used in flow cytometry or bioimaging experiments. 

### 4.6. Cell Culture

The human breast ductal carcinoma BT-474 (HTB-20™; ATCC) and MDA-MB-231 (human breast adenocarcinoma, ATCC no. HTB-26) were maintained in DMEM medium (HyClone, Logan, UT, USA) supplemented with 10% fetal bovine serum (FBS, HyClone) and 2 mM L-glutamine (PanEko, Moscow, Russia). Cells were cultured in humidified atmosphere with 5% CO_2_ at 37 °C.

### 4.7. Cell Cytotoxicity

The cytotoxicity of DARP-Lip(Bn) and EC1-LoPE were investigated using standard MTT assay [50]. Cells were seeded on a 96-well plate at 10^4^ cells per well in 100 μL of DMEM supplemented with 10% FBS and cultured overnight. Proteoliposomes DARP-Lip(Bn) or EC1-LoPE at different concentration were added to wells in 100 μL of DMEM and cells were incubated for 72 h. Then the medium was removed and MTT solution (0.5 g/L in 100 μL of DMEM) was added to the cells. Samples were incubated for 2 h at 37 °C, MTT solution was then removed and 100 μL of DMSO was added to the wells, the plate was gently shaken until the formazan crystals dissolved completely. The optical density of each well was measured using an Infinite 1000 Pro microplate reader (Tecan, Mannedorf, Zurich, Switzerland) at a wavelength of λ = 570 nm.

### 4.8. RNAse Activity

Ribonuclease activity of Bn was tested on yeast RNA by the acid-insoluble RNA precipitation assay as described in [30]. Protein solution in 40 μL buffer (0.125 M Tris-HCl, pH 8.5) was mixed with 160 μL of yeast RNA at 2 g/L and incubated at 37 °C for 15 min. The RNAse reaction was stopped by adding 200 μL of 6% HClO_3_ and incubating at 4 °C for 15 min. Undigested RNA was separated by centrifugation at 16,000 × g for 10 min at 4 °C and absorption (OD_260_) of supernatant was measured. According to [30], this value is proportional to the RNAse activity of the tested protein. 

### 4.9. Flow Cytometry

Cells (2 × 10^5^) of each cells line were treated with 50 nM protein or 0.5 nM proteoliposome dye-labeled conjugate for 10 min at 37 °C in complete medium. After that the cells were washed 3 times with PBS and analyzed with flow cytometer NovoCyte 3000 (Bioscience, San Diedo, CA, USA). Cy3.5 fluorescence was excited with a laser at 561 nm and the emission of the dye was detected using a 615/20 bandpass filter. Autofluorescence level was determined on protein-untreated cells. A minimum of 50 000 events was collected for each sample and the data were processed with NovoExpress software.

### 4.10. Measurements of Proteoliposomes Size and Zeta-Potential 

For size and zeta-potential experiments, proteoliposomes were diluted 1:1 with PBS. All the measurements were performed using a Zetasizer Nano ZS (Malvern Panalytical, Malvern, UK) at room temperature. Experiments were made in triplicate and the results are given as means ± standard deviation.

### 4.11. Tumor-Bearing Mice 

Six- to eight-week-old female BALB/c nu/nu athymic mice (22–25 g) were purchased from the SPF (specified pathogen-free) licensed nursery of the Shemyakin-Ovchinnikov Institute of Bioorganic Chemistry of the Russian Academy of Sciences. Mice were housed in specific-pathogen-free facilities with a 12-h light-dark cycle, fed with sterilized enriched laboratory food and supplied with water and food *ad libitum*. All experimental procedures were approved by the Animal Care and Use Committee of the Institute.

For tumor generation mice were inoculated subcutaneously with 2 × 10^6^ cells BT-474 stably expressing *NanoLuc luciferase* gene [51]. Inoculation of cancer cells was performed in 30% Matrigel (Corning, NY, USA) in culture medium. Tumor size was measured with Vernier calipers across two diameters every other day and the tumor volume (V) was calculated according to the formula: V = length × width^2^/2 [52].

### 4.12. In Vivo Tumor Imaging

To demonstrate the tumor-targeting capabilities of DARP-Lip(Bn) and EC1-LoPE in living animals, tumor-bearing athymic BALB/c Nude mice were administered intraperitoneal (i.p.) with proteoliposomes (10 nM) or fusion toxin (40 µg) conjugated with Cy5.5. The isoflurane inhalation for anesthesia was performed using the RAS-4 Rodent Anesthesia System (Perkin Elmer, Waltham, MA, USA). Living animal were than visualized using the IVIS Spectrum CT imaging system (Perkin Elmer, Waltham, MA, USA) with the following excitation filters: 640, 675 and 710 nm and emission filters: 680, 700, 720, 740, 760 and 780 nm, followed by Living Image software processing and autofluorescence subtraction using a Living Image Spectral Unmixing Tool. As a control of uniform settings for all acquired images, a tube containing 10 nM DARP-Lip(Bn-Cy5.5) or EC1-LoPE-Cy5.5 was imaged along with the animals.

### 4.13. Histological Analysis 

To investigate the microdistribution of DARP-Lip(Bn) and EC1-LoPE in tumors, before loading into liposomes Bn was conjugated with Cy3 and EC1-LoPE was conjugated with Cy5.5. Tumor-bearing mice were injected i.p. with EC1-LoPE-Cy5.5. DARP-Lip(Bn-Cy3) were injected i.p. 19 h later. 6 h after liposomes injection, the mice were euthanized and the tumors with the surrounding healthy tissues were excised. The fresh derived tumors were mounted on a platform with Neg-50™ frozen section medium (Richard-Allan Scientific™, Canton, MI, USA) and rapidly frozen at -26 °C. Sections of frozen tissues were performed on a ThermoFisher device using the standard method. Frozen cross sections, 15 μm thick, were placed on glass slides, air-dried and cell nuclei were additionally stained with 2 nM Hoechst 33,342 (Invitrogen, Carlsbad, CA, USA) for 10 min at 25 °C. Then the sections were washed twice with PBS, air-dried, and mounted under a cover glass in glycerol.

Sections were analyzed immediately after preparing under an LSM-710-NLO confocal microscope (Carl Zeiss, Oberkochen, Germany) using 514 nm laser for excitation Cy 3.0 and 633 nm laser for Cy 5.5. Fluorescence was detected in the range of 550–630 nm for Cy3.0 and in the range 650–740 nm for Cy 5.5. Hoechst was exited at 740 nm using femtosecond laser Chameleon Ultra II (80 MHz, 140 fs, tunable in 690–1060 nm, Coherent Inc., Santa Clara, CA, USA) and the emission was detected at 400–550 nm. To acquire the images, a 20× Plan-Apochromat (NA 0.8) objective was used.

### 4.14. In Vivo Tumor Therapy 

Twelve days after the inoculation, when tumor volumes reached ~100 mm^3^, twenty animals were randomly split into 4 groups. Treatment was carried out every other day according to the following schedules: 1) i.p. injection of 100 μL of 10 nM (~70 μg Bn) DARP-Lip(Bn), 10 doses; 2) i.p. injection of 40 μg EC1-LoPE (i.p.), 10 doses; 3) i.p. injection of 100 μL of 10 nM DARP-Lip(Bn) and 40 μg EC1-LoPE (i.p.), 10 doses; 4) i.p. injection of 100 μL PBS (control group). The tumor sizes and body weights of mice were controlled every other day. Mice were euthanized when tumors reached a volume of ∼1000 mm^3^. To calculate tumor growth inhibition coefficient (TGI), the following formula was used: TGI (%) = [(Vcontrol-Vtreatment) × 100%]/Vcontrol, where V is the tumor volume at a selected time point. 

### 4.15. Bioluminescent Imaging in Vivo

In vivo bioluminescence imaging was performed on IVIS Spectrum CT system (Perkin Elmer, Waltham, MA, USA). The BT-474/NanoLuc bearing mouse was placed in an anesthesia chamber and isoflurane inhalation was performed using the RAS-4 Rodent Anesthesia System (Perkin Elmer, Waltham, MA, USA). Furimazine was administered i.p. at a dose of 7 μg per animal, the mouse was then moved to the IVIS Spectrum CT imaging chamber, and data acquisition started one minute after the introduction of the substrate. All bioluminescence data were normalized to the acquisition conditions and are displayed in radiance (photons/s/cm^2^/str).

### 4.16. Statistics 

The data (MTT-test and tumor volume measurements) were statistically processed using one-way analysis of variance (ANOVA). 

## 5. Conclusions

The present study provides evidence that dual targeting therapy of solid tumors with protein-based toxic preparations recognizing different tumor-associated antigens is effective not only in eliminating the growth of primary tumor, but also in preventing the development of distant metastases. The proposed approach can be considered as a potential strategy with the efficacy significantly exceeding that of the mono-specific targeting therapy.
